# Neuroanatomical findings in isolated REM sleep behavior disorder and early Parkinson’s disease: a Voxel-based morphometry study

**DOI:** 10.1007/s11682-023-00815-0

**Published:** 2023-10-28

**Authors:** Giulia Donzuso, Calogero E. Cicero, Loretta Giuliano, Raffaele Squillaci, Antonina Luca, Stefano Palmucci, Antonello Basile, Giuseppe Lanza, Raffaele Ferri, Mario Zappia, Alessandra Nicoletti

**Affiliations:** 1 Department of Medical, Surgical Sciences and Advanced Technologies “GF Ingrassia”, University of Catania, Via Santa Sofia 78, 95123 Catania, Italy; 2 Radiodiagnostic and Radiotherapy Unit, University Hospital “Policlinico-San Marco”, Via Santa Sofia 78, 95123 Catania, Italy; 3 Sleep Research Center, Department of Neurology IC, Oasi Research Institute - IRCCS, Troina, Italy; 4 Department of Surgery and Medical-Surgical Specialties, University of Catania, 95123 Catania, Italy

**Keywords:** Parkinson’s disease (PD), REM sleep behavior disorder (RBD), Magnetic Resonance Imaging (MRI), Voxel-based morphometry (VBM)

## Abstract

Isolated rapid eye movement (REM) sleep behavior disorder (iRBD) is a parasomnia characterized by loss of physiological atonia of skeletal muscles with abnormal behaviors arising during REM sleep. RBD is often the early manifestation of neurodegenerative diseases, particularly alpha-synucleinopathies, such as Parkinson’s disease (PD). Both structural and functional neuroimaging studies suggest that iRBD might share, or even precede, some of the features commonly found in PD, although without a definitive conclusion. Aim of the study is to evaluate the presence of structural abnormalities involving cortical and subcortical areas in PD patients with RBD and iRBD. Patients with video-polysomnographic (VPSG)-confirmed iRBD, and patients with a diagnosis of PD were recruited. In all PD patients, the presence of probable RBD was assessed during the follow-up visits (PD/pRBD). A group of healthy controls (HC) subjects was also recruited. Each subject underwent a structural brain MRI using a 3-D T1-weighted spoiled gradient echo sequence. Twenty-three patients with iRBD, 24 PD/pRBD, and 26 HC were enrolled. Voxel-based morphometry-AnCOVA analysis revealed clusters of grey matter changes in iRBD and PD/pRBD compared to HC in several regions, involving mainly the frontal and temporal regions. The involvement of cortical brain structures associated to the control of sleep cycle and REM stage both in PD/pRBD and iRBD might suggest the presence of a common structural platform linking iRBD and PD, although this pattern may not underlie exclusively RBD-related features. Further longitudinal studies are needed to clarify the patterns of changes occurring at different time points of RBD-related neurodegeneration.

## Introduction

Rapid eye movement sleep behavior disorder (RBD) is a condition clinically characterized by episodes of dream enactment during REM sleep. Patients experience unpleasant and vivid dreams associated to complex movements and, often, vocalizations (Dauvilliers et al., 2018). These altered sleep behaviors depend on the dysfunction of the lower brainstem nuclei leading to a dyscontrol of the muscle atonia physiologically present during REM sleep, causing movements sometimes so vigorous that can lead to injury (Iranzo et al., 2016). According to the current diagnostic criteria, a definite diagnosis can be made only with a polysomnographic recording (PSG) showing the lack of atonia during REM sleep and the report of abnormal behaviors, compatible with dream enactment which may or may not be recorded by video-PSG (VPSG) («ICSD-3 Online Version—American Academy of Sleep Medicine (AASM)» s.d.) (American Academy of Sleep Medicine, 2014).

Although RBD could be considered a relative rare disorder (Cicero et al., 2021a), it has gained increased relevance in the field of neurodegenerative disease, as accumulating evidence highlighted that most cases of RBD tend to develop an alpha-synucleinopathy over the course of the years (Postuma et al., 2019) with studies describing up to 81% of patients developing an alpha-synucleinopathy after an average of 14 years follow-up (Schenck et al., 2013). Indeed, RBD has been considered to be the strongest prodromal marker in the diagnosis of prodromal Parkinson’s Disease (PD) (Berg et al., 2015).

Advanced neuroimaging techniques are a promising method to identify structural and functional features that could assess the risk of conversion toward alpha-synucleinopathies. Very recently, neuroimaging data, mostly from structural MRI, combining different techniques, have been reviewed (Campabadal et al., 2019; Ghaderi et al., 2023; Valli et al., 2022), highlighting the presence of cortical and subcortical abnormalities, involving basal ganglia and fronto-temporal cortices in iRBD patients (Unger et al., 2010, Rahayel et al., 2015, 2018a, b, Chen et al., 2022, Hanyu et al., 2012, Matzaras et al., 2022, Scherfler et al., 2011) and in PD with RBD (Boucetta et al., 2016; Jia et al., 2022; Lim et al., 2016; Rahayel et al., 2019; Salsone et al., 2014). Although PD with RBD patients had a more prominent involvement of the thalamus, putamen, insula, precuneus, para-hippocampal, and postcentral gyrus, iRBD had the internal capsule, caudate, thalamus, and fornix more involved (Campabadal et al., 2019; Valli et al., 2022). Moreover, signs of degenerative parkinsonism in iRBD were detected through the lack of the so-called dorsolateral nigral hyperintensity on high-field MRI susceptibility weighted (Marzi et al., 2016). Again, iRBD patients showed severe microstructural changes in the brainstem, right substantia nigra, olfactory area, left temporal lobe, fornix, and internal capsule (Unger et al., 2010). Alongside, few studies identified increases in GM volume (Campabadal et al., 2019; Chen et al., 2022; Holtbernd et al., 2021; Marzi et al., 2016; Rahayel et al., 2015, 2018a, b; Salsone et al., 2014; Unger et al., 2010), suggesting that the volumetric increase may reflect a compensatory phenomenon. These structural results are also corroborated by fMRI studies showing abnormalities in nigro-striatal e nigro-cortical connectivity (Ellmore et al., 2013), basal ganglia networks (Rolinski et al., 2016), cerebellum and posterior regions (Liu et al., 2021), and altered activity in the motor cortex (Li et al., 2017).

Taken together, changes in some brain areas may underlie both motor and sleep disorders; namely, iRBD might share or even precede some of the neuroimaging features commonly found in overt PD (Ghaderi et al., 2023). In this complex scenario, neuroimaging helps the understanding of the sleep disturbances associated with PD by examining the structural and functional implications of both movement and sleep disorders.

Based on these considerations, the aim of the present study was to evaluate structural changes involving GM in a group of iRBD patients and in a group of PD with probable RBD (PD/pRBD), both compared to a healthy control (HC) group. We hypothesized the presence of a possible common pattern of brain structural abnormalities occurring in these conditions, thus suggesting a similar involvement of cortical brain areas associated to the control of sleep cycle and REM stage in both PD/pRBD and iRBD.

## Methods

### Patients and clinical assessment

From 2017 to 2021, patients with iRBD were enrolled among subjects previously identified in a population-based study investigating iRBD prevalence in the communality of Catania, Italy (Cicero et al., 2021b), and among patients attending the Neurologic Clinic of the University of Catania, Italy and the Sleep Research Centre of the Oasi Research Institute-IRCCS, Troina, Italy. Diagnosis of definite iRBD was sought on the basis of a VPSG recording, using a total of six to eight EEG channels, placed according to the International 10–20 system, one or two ECG derivations, submentalis muscle EMG, bilateral flexor digitorum superficialis muscle EMG and bilateral anterior tibialis muscle EMG, EOG (two channels), nasal thermistor, snore monitor, chest and abdominal movements, pulse rate and oximetry (Micromed SpA, Mogliano Veneto, Italy). American Academy of Sleep Medicine (AASM) (American Academy of Sleep Medicine, 2014) criteria were used for sleep scoring and presence of REM sleep without atonia (RSWA) was visually assessed. RBD was diagnosed according to the ICSD-3 («ICSD-3 Online Version—American Academy of Sleep Medicine (AASM)» s.d.) (American Academy of Sleep Medicine, 2014).

In the same period, early PD patients (disease duration < 2 years) attending the “Parkinson’s Disease and Movement Disorders Centre” of the University of Catania and fulfilling the MDS-PD diagnostic criteria (Postuma et al., 2015) were consecutively enrolled. The purpose of consecutive enrolment of patients is to assure that patients who fulfil all inclusion criteria are enrolled providing a representative patients’ sample (Bjørn et al., 1998). Presence of RBD was assessed during the follow-up visits using a semi-structured interview based on the RBD screening questionnaire (RBDSQ) (Marelli et al., 2016), defining PD patients as PD/pRBD.

Neurological examination was performed by neurologists, expert in movement disorders. Motor impairment was evaluated with the Unified Parkinson’s Disease Rating Scale part-III (UPDRS-III) (Fahn et al., 1987) and the Hoehn and Yahr (HY) scale (Hoehn & Yahr, 1967). PD patients were evaluated when in “Off” state and clinical and pharmacological data were collected from the patient’s medical records. A group of healthy controls (HC) was selected among caregivers of PD and iRBD patients attending our centres. HC with probable RBD (positive at the RBDSQ and/or symptoms reported by the bed partner), subjects with a MMSE score < 24, impairment of activities of daily living and/or psychiatric disorders (i.e. depression, anxiety, panic attack, psychosis, drugs utilization, alcoholism) were excluded.

This study was carried out in accordance with the Declaration of Helsinki and approval from the local ethical committee (Ethical Committee Catania 1) was obtained. All the participants were asked to sign an informed consent prior to be included in the study.

### MRI data acquisition

Brain MRI was performed according to a routine and standardized protocol with a 1.5 T unit (Signa HDxt, and Signa Excite, GE Medical Systems, Milwaukee, WI, USA), unified for the two clinical centres. A 3D T1-weighted high-resolution spoiled gradient echo (SPGR) sequence with a 1.2-mm slice thickness and an isotropic in-plane resolution of 0.98 mm was acquired with the following parameters: repetition time 14.8 ms, echo time 6.4 ms, flip angle 25°, 115 slices, matrix size 256 X 256 and a field of view of 24 cm. Additionally, all patients underwent also a T2-weighted and FLAIR images in order to exclude morphological abnormalities, vascular disease or intracranial lesions.

### Voxel-based morphometry

We performed a voxel-based analysis investigating grey matter (GM) volume changes. Data were processed using the MATLAB R2017a and SPM8 software (http://www.fil.ion.ucl.ac.uk/spm), where we applied VBM implemented in the VBM8 toolbox (http://dbm.neuro.uni-jena.de/vbm.html) and incorporated the DARTEL toolbox that was used to obtain a high-dimensional image registration and normalization. Images were bias-corrected, tissue classified and registered using linear (12-parameter affine) and non-linear transformations, within a unified model. Subsequently, the warped GM segments were affine transformed into MNI-152 space and were scaled by the Jacobian determinants of the deformations (modulation). Finally, the modulated volumes were smoothed with a Gaussian kernel of 8-mm full width at half maximum (FWHM) (Ashburner & Friston, 2005).

### Statistical analysis

Data were analyzed using STATA 12.1 software packages (StataCorp, College Station, TX, USA). Quantitative variables were described using mean and standard deviation. Differences between means and proportions were evaluated by ANOVA or t-test and the Chi-square test, respectively.

### Image analysis

The GM volume maps were statistically analyzed using the general linear model based on Gaussian random field theory. Analysis of covariance (AnCOVA) was used for investigating the main effect of group (F-test). The advantage of an SPM-related F statistic is that changes in GM volumes are analyzed together to detect morphological changes in three or more groups. Age, gender and total intracranial volume (tICV) were included as covariates of no-interest. A conservative approach with a whole-brain statistical threshold correction [*p* < 0.05, family-wise error (FWE)] was applied; to avoid spurious results a cluster threshold comprising at least 50 voxels was considered. Post hoc t-tests were performed to identify GM changes between each group, setting a statistical threshold at the voxel level at *p* < 0.05 FWE and using the small volume corrections (*p* < 0.05 FWE-SVC), by centering a 3 mm sphere around the cluster peak coordinates.

## Results

### Demographics and clinical data

Twenty-three patients with iRBD (17 men; age 64.2 years, 10.8 SD) confirmed with VPSG, and 24 patients with PD/pRBD with a short disease duration and mild stage of disease (14 men; age 63.0 years, 6.2 SD) were enrolled for the study. Twenty-six age- and sex-matched HC (14 men; mean age 59.3 years, 7.8 SD) were also enrolled (Table 1). Age and sex were not significantly different between groups.

**Table 1:** Demographics and clinical characteristics of PD patients, iRBD patients and healthy controls

	1. PD/pRBD	2. iRBD	3. HC	*p-value*		
	*n* = 24	*n* = 23	*n* = 26	*1 vs 2*	*1 vs 3*	*2 vs 3*
Gender (men)	14 (58.3%)	17 (73.9%)	14 (53.8%)	0.25	0.75	0.14
Age (years)	63.0 ± 6.2	64.2 ± 10.8	59.3 ± 7.8	0.63	0.07	0.07
Age at onset (years)	61.2 ± 6.3	/	/	/	/	/
Disease duration (years)	1.7 ± 1.5	/	/	/	/	/
UPDRS-ME score	28.6 ± 9.9	/	/	/	/	/
Hoehn-Yahr stage	2.0 ± 0.3	/	/	/	/	/

Data are given as means ± standard deviations. *PD/pRBD* Parkinson’s Disease with probable REM sleep behavior disorder; *iRBD* isolated REM sleep behavior disorder. *HC* healthy controls; *UPDRS-ME* Unified Parkinson’s Disease Rating Scale-Motor Examination

### GM changes across and between groups

VBM analysis, investigating the neuroanatomical changes occurring when the three groups were analyzed together (AnCOVA, F-test), showed the presence of six clusters of GM abnormalities involving right orbitofrontal and superior frontal gyrus, lingual, fusiform gyri, and bilateral precentral gyrus at *p* < 0.05 FWE at cluster level (Table 2).

**Table 2:** AnCOVA analysis showing global maxima of clusters of significantly abnormal grey matter volume in PD/pRBD, iRBD and healthy controls

Brain Regions	Hemisphere	Cluster size	Peak activation*	*p*-value	Peak coordinates		
*Covariates: gender, age, tICV*					x	y	z
Orbitofrontal gyrus	right	97	14.74	< 0.001	15	28	-14
Lingual gyrus	right	149	14.54	< 0.001	15	-64	-2
Precentral gyrus	right	161	12.92	0.001	48	-3	34
Precentral gyrus	left	67	9.44	0.004	-20	-15	69
Fusiform gyrus	right	52	9.74	0.004	34	-3	-30
Superior frontal gyrus	right	80	9.48	0.004	26	42	18

*PD/pRBD* Parkinson’s Disease with probable REM sleep behavior disorder; *iRBD* isolated REM sleep behavior disorder. The coordinates x, y and z refer to the anatomical location, indicating standard stereotactic space as defined by Montreal Neurological Institute. *F-value; *p* < 0.05 FWE, cluster size > 50 voxels

**Fig. 1:**
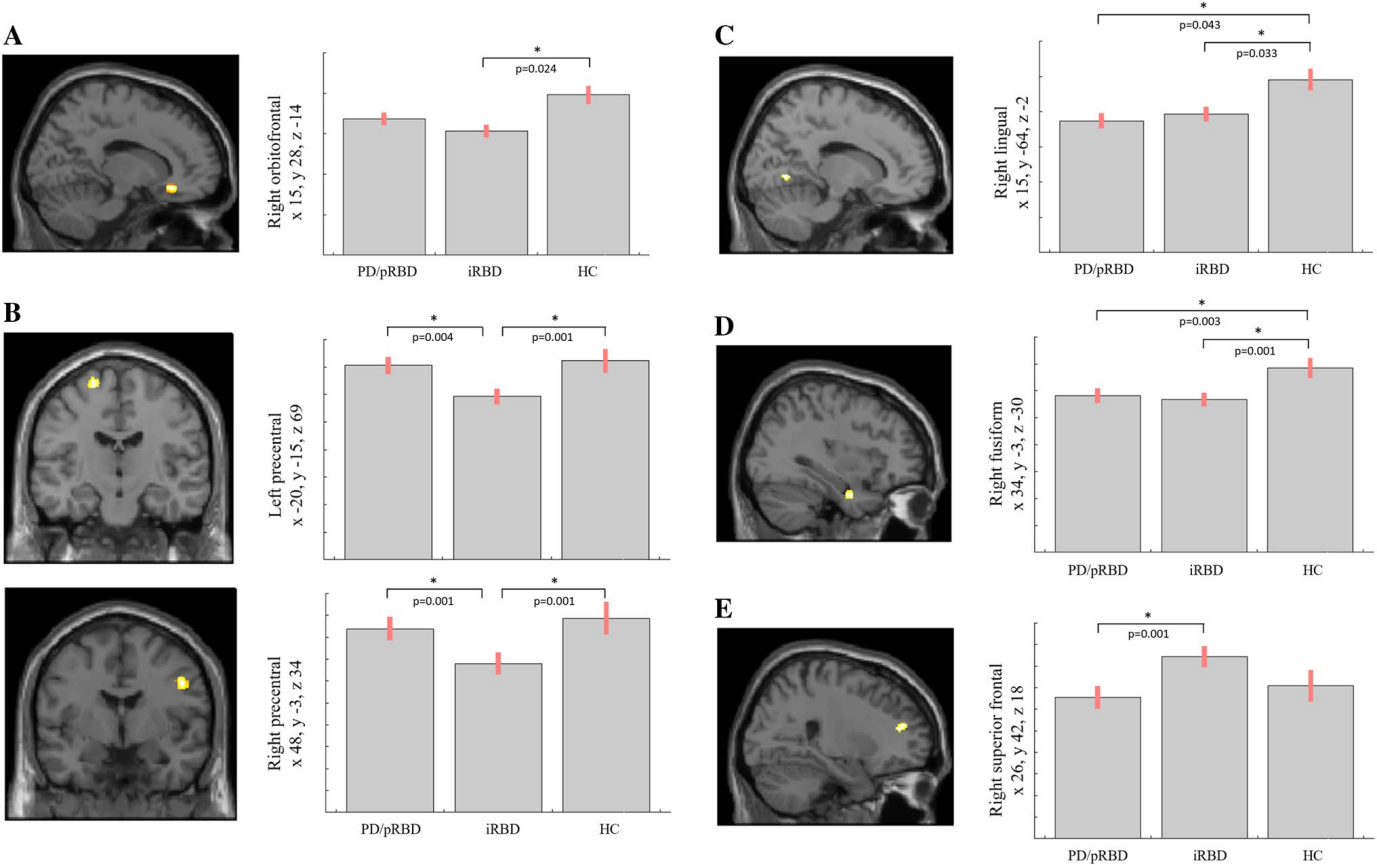
Significant clusters of grey matter changes in the *post-hoc* t-test analysis. **A** right orbitofrontal. **B** left and right precentral. **C** right lingual. **D** right fusiform. **E** right superior frontal. *Statistical threshold at *p* < 0.05 FWE, cluster size > 50 voxels. PD/pRBD = Parkinson’s Disease with probable REM sleep behavior disorder; iRBD = isolated REM sleep behavior disorder; HC = healthy controls

Post hoc t-test analysis revealed the difference between each group pair, setting statistical threshold at *p* < 0.05 FWE-SVC. iRBD subjects showed a statistically significant decrease of GM volume involving the right orbitofrontal gyrus compared to HC (t = 5.43, local maxima: × 15, y 28, z -14, p-value 0.024) (Fig. 1A), and bilateral precentral gyrus compared both to PD/pRBD and HC (right, t = 4.35, local maxima: × 48, y -3, z 34, p-value 0.001 iRBD *vs* PD/pRBD, p-value 0.001 iRBD *vs* HC; left t = 3.60, local maxima: x -20, y -15, z 69, p-value 0.004 iRBD *vs* PD/pRBD, p-value 0.001 iRBD *vs* HC) (Fig. 1B); clusters of decreased GM volume including right lingual gyrus (t = 4.17, local maxima: × 15, y -64, z -2, p-value 0.043 PD/pRBD *vs* HC, p-value 0.033 iRBD *vs* HC) (Fig. 1C) and fusiform gyrus (t = 4.27, local maxima: × 34, y -3, z -30, p-value 0.003 PD/pRBD *vs* HC, p-value 0.001 iRBD *vs* HC) (Fig. 1D) were detected in PD/pRBD and iRBD patients compared to HC. A cluster of increased GM volume in the right superior frontal gyrus was present in iRBD patients when compared to PD/pRBD (t = 4.31, local maxima: × 26, y 21, z 18, p-value 0.001) (Fig. 1E). No other significant differences between groups were found.

## Discussion

### Main findings

In agreement with previous literature (Chen et al., 2022; Jia et al., 2022; Matzaras et al., 2022; Rahayel et al., 2019), our study showed the presence of decreased GM volume in frontal areas (right orbitofrontal cortex and bilateral precentral gyrus) in iRBD patients compared to both PD/pRBD and HC, and in temporo-occipital regions (lingual and fusiform gyri) in both iRBD and PD/pRBD patients in comparison with HC. Furthermore, we also reported the presence of increased GM volume in superior frontal gyrus in iRBD patients compared to PD/pRBD.

Several studies have been conducted investigating GM changes in iRBD patients and/or PD with RBD, often reporting heterogeneous findings (Campabadal et al., 2019; Ghaderi et al., 2023; Valli et al., 2022). Decreased GM volume have been reported in different brain areas, involving anterior (frontal cortex) and posterior (parieto-occipital cortices) regions, in both iRBD (Chen et al., 2022; Matzaras et al., 2022) and PD/RBD patients (Jia et al., 2022; Rahayel et al., 2019), but, on the other hand, increased GM volume involving cortical and subcortical regions has also been reported, suggesting compensatory mechanism (Chen et al., 2022; Holtbernd et al., 2021). However, differences in neuroimaging approaches, clinical characteristics of the enrolled patients (definite *versus* probable iRBD, early *versus* advanced PD etc.) limit the comparison across the studies.

Frontal involvement in iRBD, including orbitofrontal and precentral cortices, has been demonstrated in several studies (Chen et al., 2022; Rahayel et al., 2018a, b). In particular VBM findings have shown a reduced GM volume in iRBD compared to HC in the motor loop, including frontal lobes and these reductions were associated with lower motor performance and clinical iRBD manifestations (Rahayel et al., 2018a). It is well known that iRBD is a major risk factor for PD development (Postuma et al., 2013); the pattern of these structural abnormalities strongly overlaps with cortical regions known to support motor abilities and might suggest that abnormalities in the motor-related cortical areas may already be present before the parkinsonism clinical onset. This hypothesis is also supported by functional MRI studies that have shown abnormalities in the frontal and prefrontal networks in iRBD subjects (Wakasugi et al., 2021), consistent with an early subtle involvement of executive dysfunction providing a promising early biomarker of both cognitive and motor network dysfunctions of alpha-synucleinopathies.

Nonetheless, in our sample we did not find any significant differences in the precentral gyrus between PD/pRBD and HC. Notably, it should be noted, that also previous studies have shown no differences in cortical regions between PD/RBD and HC (Rolinski et al., 2016; Salsone et al., 2014). Although we have not a clear explanation, these findings could be probably due to the intact cognitive status and the short disease duration of the included patients. As a matter of fact, literature on GM atrophy in early PD is divergent, and MRI studies do not demonstrate a consistent pattern (Banwinkler et al., 2022).

More consistent findings have been reported in literature concerning the involvement of the posterior regions in both iRBD and PD/RBD (Campabadal et al., 2019; Guo et al., 2018; Rahayel et al., 2015, 2019; Unger et al., 2010).

In agreement with literature, we found in both iRBD and PD/pRBD a temporal cortex atrophy involving the fusiform gyrus. Decreased cortical thickness in temporal cortices, including fusiform area, has been found in PD/RBD compared to PD patients without RBD (Rahayel et al., 2019) and in iRBD compared to HC (Campabadal et al., 2019; Rahayel et al., 2015, 2018a, b; Unger et al., 2010). Accordingly, a recent voxel-wise metanalysis including VBM studies, showed a significantly reduced GM volume in the right superior temporal gyrus in PD/RBD, compared to PD without RBD, suggesting the association between the occurrence of RBD in PD patients and atrophy in the temporal areas (Yang et al., 2020). In agreement with these findings, perfusion and metabolism imaging studies demonstrated the presence of changes in metabolism in temporal areas, in RBD patients compared to HC (Ge et al., 2015; Mazza et al., 2006; Vendette et al., 2011). The involvement of temporal areas in RBD and the underlying mechanisms remain to be understood. Temporal and limbic areas are found to be significantly activated during physiological REM sleep (Nir & Tononi, 2010); thus, these abnormalities could reflect a disruption of the normal atonia circuitry leading to the occurrence of RBD behaviors. On the other hand, temporal GM changes could be associated to the RBD-related clinical characteristics of PD patients, including cognitive impairment, spatial information processing and mood abnormalities (Yang et al., 2020). The presence of a similar pattern of structural abnormalities involving temporal regions in iRBD and PD/RBD groups has been previously faced by Pereira and coll (Pereira et al., 2019) suggesting the presence of a high prevalence of future iRBD to PD converters in their sample, meaning that subtle changes were already present in patients with iRBD (Pereira et al., 2019).

Our data also show the presence of decreased GM volume in the posterior regions, involving the lingual gyrus in PD/pRBD and iRBD subjects, compared to HC. Abnormalities in the occipital regions have previously been demonstrated in PD/RBD (Campabadal et al., 2019; Guo et al., 2018; Rahayel et al., 2019). The occipital and frontal connections with the brainstem reticular formation, a core pathological region in RBD, are well known (Jang & Kwon, 2015). Thus, damage in the brainstem reticular formation may cause a potential effect on structures and networks involving the occipital and frontal cortex, to rebalance the abnormalities in the reticular formation (Guo et al., 2018).

Considering all these results, iRBD subjects seem to show a greater involvement of frontal cortical regions than PD/pRBD (bilateral precentral and right orbitofrontal gyri) and to share similar posterior cortical abnormalities with PD/pRBD compared to HC (right fusiform and lingual gyri). As previously demonstrated (Campabadal et al., 2019; Mazza et al., 2006), an anterior (i.e., the orbitofrontal cortex) and posterior (i.e., the parieto-occipital and temporal cortices) pattern of cortical atrophy was consistently described in iRBD subjects (Valli et al., 2022). Taking into account the degree of cortical involvement, we cannot exclude that this could be due to the different RBD duration of iRBD subjects with respect to that of PD/pRBD, as well as to the presence of subjects in the prodromal stages of different synucleinopathies in the iRBD cohort that may convert overtime.

As for the GM volume increase involving superior frontal gyrus in iRBD patients compared to PD/pRBD, this finding is consistent with previous studies reporting the occurrence of volume increases involving cortical (Park et al., 2019) and subcortical regions (Holtbernd et al., 2021), as well as of metabolic increases in frontal cortex by means of the FDG-PET in iRBD patients (Kim et al., 2021). The authors suggest that cortical activations of the frontal areas might produce overactivity in the subcortical regions, such as the caudate nucleus, thus resulting in the expression of excessive movement during sleep in iRBD subjects (Park et al., 2019). Accordingly, the presence of hypermetabolism in the frontal regions, together with hypometabolism in the posterior regions (both representing areas of structural and functional changes), might reflect a co-occurrence of neurodegenerative and compensatory phenomena (Kim et al., 2021). These findings are in agreement with structural/functional data showing changes in connectivity values involving occipital cortices together with the presence of decreased GM volume than HC, and increases in cerebellum and deep grey nuclei (Chen et al., 2022). Therefore, the difference between iRBD and PD/pRBD may be explained by a continuum ranging from “pure” iRBD to a more pronounced brain abnormality as seen in overt PD (Chen et al., 2022; Holtbernd et al., 2021).

Finally, it is interesting to note that most of the present MRI findings affected the right hemisphere, although the occurrence of less significant results in the left hemisphere does not necessarily imply that the left hemisphere is not involved. Nevertheless, the threshold established for statistical analysis might contribute to explain these data. Notably, an increased frequency of RBD (Sommerauer et al., 2014) and poorer spatial memory performances have been associated to a left-side onset of the disease (Foster et al., 2008), thus suggesting an involvement of the right hemisphere.

### Strengths and limitations

Strengths of our study are the inclusion of three groups considering also healthy controls and the presence of VPSG confirmation for iRBD subjects together with the high statistical threshold FWE-corrected for imaging analysis.

However, some limitations of our study must be acknowledged. First, the lack of VPSG confirmation for PD patients; as we specified above, the diagnosis of PD/pRBD was confirmed during the follow-up visits only using a semi-structured interview based on the RBDSQ, representing a useful tool for detecting RBD in PD patients (Nomura et al., 2011). Another limitation to consider is the relatively small sample size, that could affect the statistical power of our analysis, but the strength of statistical threshold and cluster size minimize this risk. An additional caveat is the use of a 1.5 T scanner to acquire MRI data, which may represent a possible limitation in comparison to recent studies using higher MRI fields. Finally, considering that iRBD is a clinical model of prodromal alpha-synucleinopathies and given that this study design did not include a group of PD patients without RBD, the interpretation of the present results, at least at this stage, is challenging. Currently, indeed, most of these findings might be interpreted as structural brain atrophy associated with PD at a prodromal/early stage of the disease, probably associated with different subtypes of evolution, and not specifically associated to RBD symptomatology. Furthermore, we do not have data about RBD duration in PD/pRBD patients that might have been of interest in the further interpretation of data based on the “brain-first vs. body-first” theory.

### Conclusions

The involvement of cortical brain structures associated to the control of sleep cycle and REM stage both in PD/pRBD and iRBD might suggest the presence of a common structural platform linking iRBD and PD, although this pattern may not underlie exclusively RBD-related features; furthermore, the occurrence of bidirectional changes (decrease and increase GM volume) of the structural integrity in different brain regions suggests a possible concomitant occurrence of both compensatory and neurodegenerative phenomena. Further longitudinal imaging studies are needed to clarify the pattern of changes occurring at different time points and to identify early neuroimaging features of neurodegeneration in iRBD patients.

## Data Availability

The data that support the findings of this study are available from the corresponding author upon reasonable request.
